# Excitation–emission matrix fluorescence spectroscopy for cell viability testing in UV-treated cell culture†

**DOI:** 10.1039/d1ra09021f

**Published:** 2022-03-09

**Authors:** Klaudia Głowacz, Sandra Skorupska, Ilona Grabowska-Jadach, Patrycja Ciosek-Skibińska

**Affiliations:** Chair of Medical Biotechnology, Faculty of Chemistry, Warsaw University of Technology Noakowskiego 3 00-664 Warsaw Poland pciosek@ch.pw.edu.pl

## Abstract

Monitoring of cells viability is essential in a number of biomedical applications, including cell-based sensors, cell-based microsystems, and cell-based assays. The use of spectroscopic techniques for such purposes is especially advantageous since they are non-invasive, label-free, and non-destructive. However, such an approach must include chemometric analysis of the data to assess the information on cells viability. In the presented article we demonstrate, that excitation–emission matrix (EEM) fluorescence spectroscopy can be applied for reliable determination of cells viability due to the high correlation of EEM fluorescence data with the MTT test data. A375 cells (malignant melanoma) were exposed to UV radiation as a physical stress factor, resulting in a decrease of viability up to *ca.* 20%, confirmed by the standard MTT test. They were also characterized by means of EEM fluorescence spectroscopy coupled with unfolded partial least squares (UPLS) regression. Statistical evaluation revealed high accordance of the two methods of viability testing in terms of accuracy, precision, and correlation. The presented results are very promising for the development of spectroscopic soft sensors that can be applied for drug screening, biocompatibility testing, tissue engineering, and pharmacodynamic studies.

## Abstract 2

Excitation-emission matrix fluorescence spectroscopy can be applied for label-free and non-destructive determination of cells viability, which is promising methodology for drug screening, biocompatibility testing, or pharmacodynamic studies.

## Graphical abstract

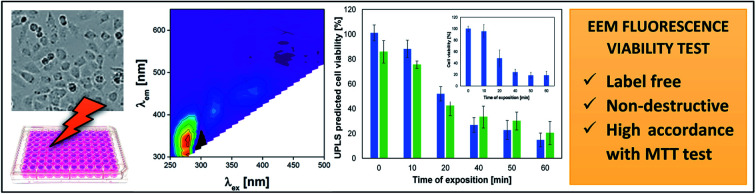

## Introduction

Monitoring the metabolic activity of cells is extremely important in many biomedical applications, from basic research to advanced pharmacological studies (*e.g.*, drug screening).^1^ Traditionally, the assessment of cell culture parameters and their changes can be determined mainly optically – with the use of microscopes, turbidimetry/nephelometry, or fluorescence, by counting cells and determining the degree of confluence of the cells suspension (determination of optical density, differential fluorescent staining, *etc.*).^2^ The viability of the cells and their ability to proliferate are determined based on counting the cells. The change of these parameters under the influence of the induced (bio)chemical factor (*e.g.*, a drug) validates its biological activity. In this way, the impact of many biologically active substances is assessed *in vitro*.^3^

There are many different tests used in *in vitro* studies to assess the condition of cell culture. The most frequently analyzed parameters when performing cytotoxicity tests are cells viability, metabolic activity, proliferation rate. Cells viability can be evaluated based on differential staining with calcein-AM (penetrates the cell membrane) and propidium iodide (stains DNA and RNA inside cells with reversibly damaged membranes). The use of two fluorescent dyes makes it possible to distinguish between live and dead cells. The most commonly used test for examination of cells metabolic activity is MTT^4,5^ or its variants (XTT, MTS). This colorimetric assay is based on the reduction of a tetrazolium salt (MTT) by metabolically active cells. The higher the absorbance of the solution, the greater the number of metabolically active cells (which corresponds to the number of viable cells). The main disadvantage of the tests described above is that they lead to cell death, so they do not allow to continue the experiment and monitor the condition changes of the cell culture over time. Therefore, there is a need to develop simple and easy to perform tests that can determine the viability of cells without terminating the experiment.

In the last few years, alternative methods for viability testing have been proposed to enable continuous control of the state of cells *in vitro* and in real-time – from impedance, through conductometric and amperometric methods, to optical sensors and biosensors.^6^ The use of electromagnetic spectroscopic techniques coupled with chemometric data analysis to monitor the cell culture is especially advantageous since they are non-invasive and non-destructive. In addition, such an approach can be realized without the necessity of labels and with little-to-no sample preparation, which makes online and real-time monitoring possible.^7^ Raman spectroscopy^8–10^ and infrared spectroscopy^11,12^ seem to be one of the most extensively studied techniques in this respect. A technique that has also started to gain attention is excitation–emission matrix fluorescence spectroscopy (EEMFS, 2D fluorescence). EEM fluorescence spectroscopy is a modern, non-invasive analytical technique consisting of recording entire emission spectra at multiple excitation wavelengths.^13^ The result of such measurement is an excitation–emission matrix (EEM, “fluorescence landscape”), a characteristic and unique signature of the tested sample, which encodes information on all fluorescent substances present in it, both in terms of their type and concentration.

Excitation–emission matrix fluorescence spectroscopy is most often used for bioprocess monitoring,^13–15^ tracing the contamination in the water samples,^16,17^ and foodstuff quality control.^18,19^ Moreover, several reports regarding the use of this multi-wavelength technique in biomedical applications can be found in the literature. For example, Dramićanin *et al.* showed that excitation–emission matrix fluorescence spectroscopy coupled with a support vector machine (SVM) allows distinguishing between normal and tumor breast tissue.^20^ Another work by de Oliveira Neves *et al.* also proves that internal biomolecular signatures contained in the excitation–emission matrix of cell culture might be utilized to identify normal and tumor cells.^21^ In the case of biological samples, including cell cultures and tissues, signals from such bioanalytes as amino acids, oligopeptides, structural proteins, enzymes, vitamins, lipids, porphyrins, *etc.*, can be observed utilizing EEM fluorescence spectroscopy.^20,22^ Then, using appropriate chemometric modeling, the extraction of the relevant information that can be related to, *e.g.*, cell state^23^ is possible. Other methodologies based on autofluorescence imaging of endogenous fluorophores present in cells or tissues show that tracking the changes in their content can be used to study cell metabolism,^24^ detect genetic mutations in cancer cells or identify stem cells subpopulations.^25,26^ Therefore, the use of excitation–emission matrix fluorescence spectroscopy coupled with multivariate data analysis can be a promising approach for assessing the most critical parameters of cell culture, such as cells viability.

**Fig. 1:**
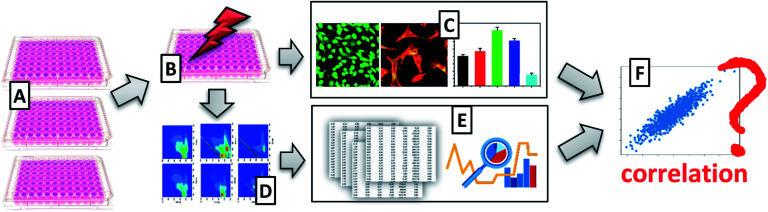
Schematic representation of experimental set-up. (A) Preparation of cell culture; (B) treatment of the cells with UV radiation; (C) assessment of cells viability with the use of classical techniques, *i.e.*, microscopy and MTT viability assay; (D) acquisition of excitation–emission matrix fluorescence data; (E) EEM fluorescence data analysis; (F) verification of correlation of EEM fluorescence data with MTT tests.

The aim of this work is assessing of cells viability with the use of excitation–emission matrix fluorescence spectroscopy. We would like to check whether this multispectral technique allows obtaining spectra correlating with viability results obtained using the MTT test, the “golden standard” in cells viability testing (Fig. 1). The proposed methodology has superior advantages over traditionally applied techniques, such as the possibility of use in real-time, in an entirely non-invasively manner, and label-free.

## Experimental

### Reagents and materials

Skin tumor cell line was used in our experiments: A375 (malignant melanoma), purchased from ATCC (Manassas, VA, USA). DMEM high glucose, penicillin and streptomycin, l-glutamine, phosphate buffer saline (PBS), and fetal bovine serum (FBS) were obtained from Biowest (Nuaillé, France). Trypple Express was purchased from Gibco (Waltham, MA, USA). MTT salt was from Sigma-Merck (Poznań, Poland), and dimethylsulfoxide (DMSO) was obtained from POCH (Gliwice, Poland).

**Fig. 2:**
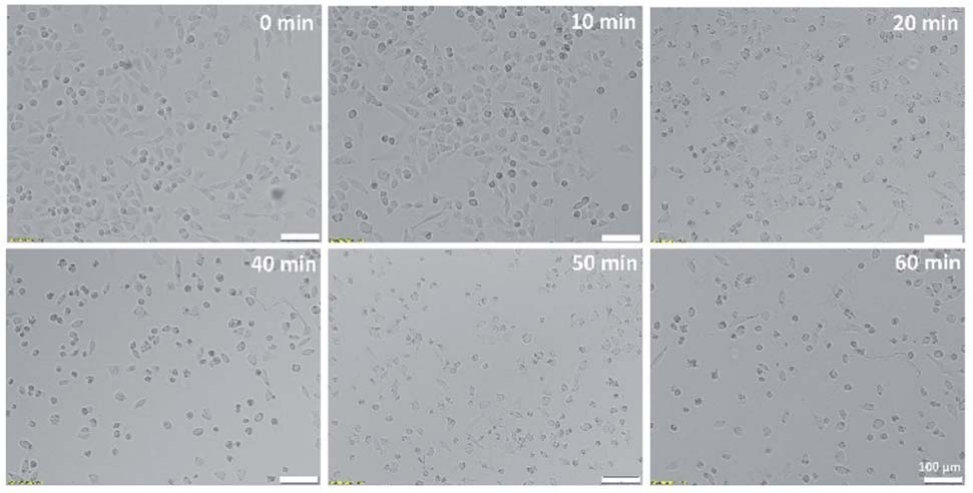
Microscopic pictures of A375 cells exposed to UV radiation of different time duration (0, 10, 20, 40, 50, 60 min).

The UVP BLAK-RAY B-100AP high intensity UV lamp was used for cell culture irradiation (Analytik Jena US, Upland, CA). Observation of changes in cells morphology (Fig. 2) was carried out using an inverted fluorescence microscope (Olympus, Center Valley, PA, USA). The absorbance measurements of the solutions in the MTT assay were performed using a multi-well plate reader Cytation-3 (BioTek, Instruments, Inc., Winooski, VT, USA).

### Cell culture and reference data determination

A375 cell line was cultured in DMEM high glucose base supplemented with 10% FBS, 1% penicillin and streptomycin, and 1% l-glutamine. Cells were subcultured every 2 days. Briefly, the medium was removed, and cells were rinsed with 1 mL of PBS. After that, Tryple Express was added (1 mL) to detach the cells from the surface of the culture vessel. Next, the cells suspension was transferred to the falcon, and cells were centrifuged for 3 min using 2000 rpm. The obtained pellet was suspended in the cell culture medium to obtain the appropriate cells density – 1.5 × 105 cells per mL. Then cells were seeded into a 96-well plate at a volume of 100 μL per well. After 24 h, cells were irradiated with UV radiation (*λ* = 365 nm) of different time duration (0, 10, 20, 40, 50, 60 min). Immediately after that, the cell medium was exchanged, and cells were incubated for the next 24 h. Cells prepared in this way were used for fluorescence analysis and cells viability test.

Cells viability was determined by the MTT assay. For this purpose, the medium was removed from the wells, and 100 μL of 0.5 mg mL^−1^ MTT salt solution was added. Cells were placed in an incubator for 4 h. Then, the solution was gently removed from the wells, and 100 μL of DMSO was added to dissolve the formazan crystals. In the next step, the absorbance of the solution was measured at a wavelength of 570 nm. Cells viability was calculated as the ratio of the mean absorbance of the test samples to the mean absorbance of the control samples that were not exposed to UV radiation.

### The collection of EEM fluorescence data

**Fig. 3:**
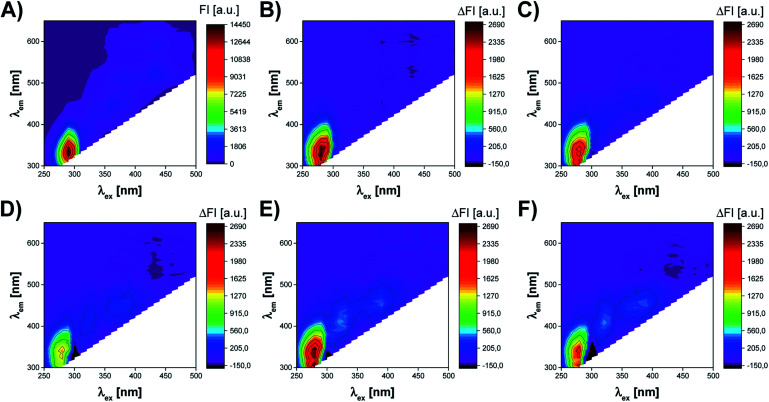
The influence of UV radiation on excitation–emission matrix (EEM) of A375 cells. (A) EEM of A375 cells not subjected to UV radiation. EEM difference spectra of A375 cells exposed to UV radiation for (B) 10 min; (C) 20 min; (D) 40 min; (E) 50 min; (F) 60 min. The difference spectra were obtained by subtracting the EEM spectrum of A375 cells not subjected to UV radiation from the EEM spectrum of A375 cells exposed to UV radiation of different time duration.

The fluorescence measurements of UV irradiated cell culture were performed by Synergy™ Neo 2 Hybrid Multi-Mode Microplate Reader fluorescence spectrometer (BioTek Instruments, Inc., Winooski, VT, USA). The acquisition of excitation–emission matrices (EEMs) was realized using a hand-written measurement protocol in which subsequent emission spectra were recorded at decreasing excitation wavelength from 500 nm to 250 nm (with 10 nm interval). The range of the recorded emission spectra depended on the excitation wavelength at which the spectrum was measured. Thus, for *λ*_ex_ in a range of 290–500 nm, the emission was recorded from *λ*_em_ = *λ*_ex_ + 20 nm to 650 nm. In the case of *λ*_ex_ <290 nm, the emission was recorded over the spectral range of 300–650 nm (Fig. 3). This method of EEM spectra acquisition allowed to avoid Rayleigh and Raman signals in the obtained spectra. The resolution of all emission spectra was 5 nm. All fluorescence measurements were carried out at 37 °C, using bottom optics and Corning® 96-well High Content Imaging Plate (Corning 4517, Corning, Inc., Corning, NY, USA). Each sample, treated by UV radiation of different time duration (0, 10, 20, 40, 50, 60 min), was measured in 12 replicates.

### Data analysis

The conducted fluorescence measurements resulted in the collection of 26 emission spectra per sample, which were further arranged in the excitation–emission matrix. As a result, each sample was described by EEM of size 26 × 71 (*λ*_ex_ × *λ*_em_). Unfolded partial least squares (UPLS) regression was used to verify whether the information encoded in the obtained EEMs can be correlated with cells viability determined with the MTT test. Therefore, before PLS modeling, each excitation–emission matrix was unfolded into a data vector by combining the excitation and emission mode (1 × [*λ*_ex_ × *λ*_em_]). Since the recorded emission range depended on the excitation wavelength at which the spectrum was acquired, the EEMs contained missing data. These missing values were omitted during the unfolding of EEMs, and the final data vector of 1 × 1340 was obtained per sample. The unfolded EEMs of all samples were then disposed of in a matrix arrangement (samples × [*λ*_ex_ × *λ*_em_]) and subjected to further data analysis. Autoscaling was applied as a preprocessing method. The chemometric analysis was performed in Solo (Eigenvector Research Inc., Manson, US), while the statistical evaluation of the results was conducted using MS Excel (Microsoft, Redmond, US) software. The figures were generated with Origin (OriginLab Corporation, Northampton, MA, USA) or MS Excel (Microsoft, Redmond, US) software.

## Results and discussion

### Effect of UV radiation on A375 cells

**Fig. 4:**
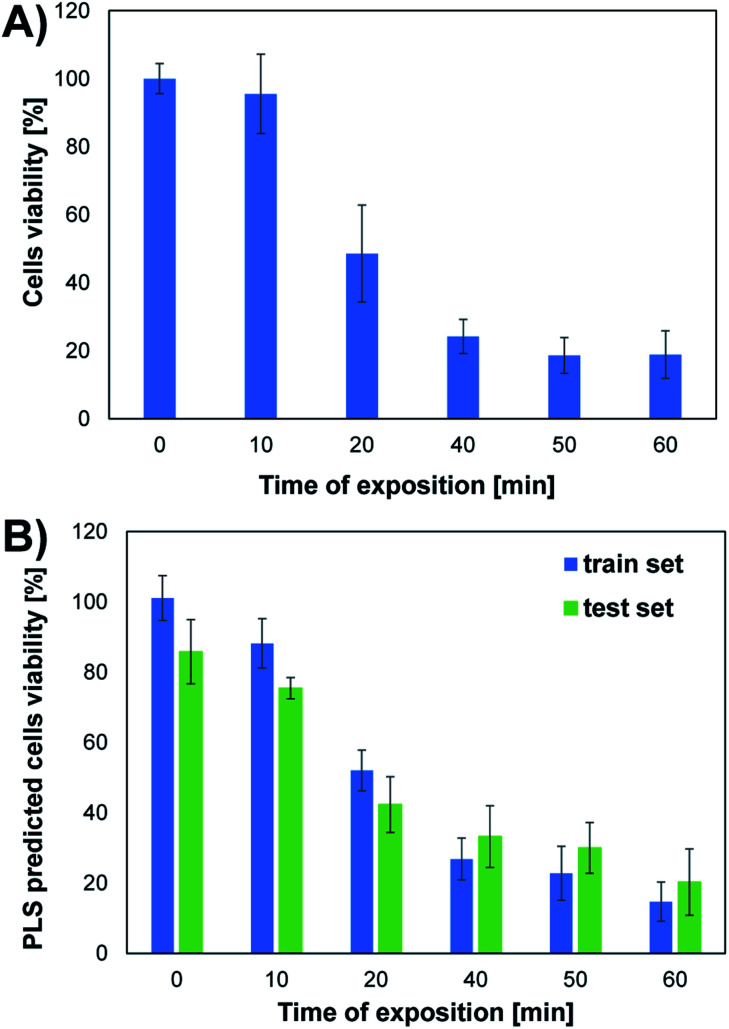
Viability of A375 cells (mean ± SD, *n* ∈ [3,9]) exposed to UV radiation of different time duration. (A) Cells viability determined by MTT test; (B) UPLS predicted cells viability for train and test set.

The conducted studies assessed changes in cells viability caused by the action of UV radiation. The choice of such a physical factor was dictated by the fact that our skin is constantly exposed to UV radiation. This kind of radiation is emitted by the sun and artificial sources. UV radiation may have a negative effect on the cells of living organisms. It can contribute to the formation of free radicals and DNA damage, which can even lead to cell death. As part of the research, the effect of UV radiation on the viability and morphology of A375 skin cancer cells was evaluated. For this purpose, A375 cells exposed to UV radiation for various time were observed under a microscope, and the MTT cells viability assay was performed. Fig. 2 shows the microscope images of cells 24 h after UV irradiation. Depending on the time of irradiation of cells (in the range of 10–60 min), changes in cells' density and morphology can be noticed. In the case of UV irradiation of cells for 10 min, no changes in cell morphology and cells density were observed compared to the control sample, which was not exposed to UV radiation. In the 20 minute irradiation test, a much larger number of shrunken, round cells detached from the vessel's surface was noticed. In the case of cell culture treated with UV radiation 40 min and above, a much lower cell density was observed due to the detachment of cells from the surface of the vessel and their death. Most of the cells in the field of view have a shrunken, oval shape, which also indicates the start of the cell death process. The microscopic observations are consistent with the MTT cells viability assay results. As shown in Fig. 4A, cells viability after 10 min of UV irradiation is at a very high level. A decrease in the viability of the cells to approx. 50% was observed after exposure to UV radiation for 20 min. In the case of cell culture irradiated with UV for 40 min and longer, the cells viability is very low and amounts to less than 25%. There is a clear relationship between the time of exposure to UV radiation and cells viability.

### Unfolded PLS analysis of EEM data

The cells of the living organism contain several endogenous fluorophores, which are involved in cellular growth and metabolic activity. Therefore, the monitoring of the presence and changes in the content of compounds such as fluorescent amino acids (tryptophan, tyrosine, phenylalanine), cofactors (NADH, NADPH, FAD, FMN), vitamins (riboflavin, pyridoxine), lipids, and porphyrins may allow the assessment of the most crucial parameters of cell culture.^20,22,25^ Excitation–emission matrix fluorescence spectroscopy can be utilized to capture the information on all these fluorophores, which content directly or indirectly reflects the state of cell culture. Thus, this work aimed to verify if fluorescent fingerprints (excitation–emission matrices) of cell culture contain the information, which might be useful to assess the viability of the cells.

Fig. 3 presents the effect of UV radiation on the excitation–emission spectrum of A375 cells. As shown in Fig. 3A, the control sample of A375 cells exhibits fluorescence at several spectral regions (around *λ*_ex_/*λ*_em_: 290 nm/340 nm, 360 nm/440 nm, 390 nm/500 nm, 390 nm/590 nm, 430 nm/520 nm, and 430 nm/590 nm), which may be associated with endogenous fluorophores present in cells. However, the cell medium itself is a complex chemical mixture also containing fluorescent compounds, *e.g.*, fluorescent amino acids and vitamins.^27^ Therefore, it is difficult to capture the subtle changes induced by UV radiation in the raw excitation–emission matrices. To better visualize differences between EEMs of A375 cells irradiated for 10–60 min, the difference spectra were prepared by subtracting control from samples exposed to the stress factor (Fig. 3B–F). The analysis of EEM difference spectra reveals that there are 3 main spectral regions affected by the action of UV radiation. First of all, the slight, irregular decrease of the fluorescence intensity is observed at *λ*_ex_ ∈ (250 nm, 300 nm), *λ*_em_ ∈ (300 nm, 420 nm). The differences in the fluorescence signal in this region are most probably attributed to the changes in the content of aromatic amino acids, *i.e.*, tyrosine and tryptophan.^13,22^ In addition, DNA also fluoresces in this spectral range.^28^ As the UV irradiation time of A375 cells extends, the increase of fluorescence signal in the ranges of *λ*_ex_ ∈ (300 nm, 340 nm), *λ*_em_ ∈ (360 nm, 460 nm) and *λ*_ex_ ∈ (340 nm, 430 nm), *λ*_em_ ∈ (420 nm, 480 nm) can also be noticed. Typically, in biological samples, changes in these regions of the fluorescence spectrum correspond to the presence of fluorophores such as coenzymes and vitamins.^13^ It is worth noticing that after 10 min of UV irradiation, there are the least visible differences in the EEM spectrum compared to the control sample (Fig. 3B). This observation is consistent with the microscopic image of A375 cells and the results of the MTT assay, which prove a negligible impact of 10 min UV irradiation on the viability of the cells (Fig. 2B and 4A). After 20 and more minutes of exposure of A375 cells to the stress factor, a significant decrease of cell viability is observed (Fig. 4A). For these time points, the most substantial changes in the excitation–emission matrices of A375 cells can be noticed, indicating that the cells viability's decrease directly impacts the obtained fluorescence landscape (Fig. 3C–F).

The employment of spectroscopic techniques to monitor cell culture parameters is most often based on a pattern-based sensing approach, in which non-selective analytical signals are used to describe the biological sample. The obtained spectroscopic spectra (so-called “chemical fingerprints”) are potentially rich in chemical information, as it contains inputs from all individual components of the sample. Therefore, due to complex chemical composition, multivariate data analysis methods are necessary to extract the relevant information from such spectra.^10–13^ In this work, unfolded partial least squares (UPLS) regression was used to explore the relationship between the information encoded in excitation–emission matrices of UV irradiated A375 cells with cells viability obtained using the MTT test (reference data). Unfolded partial least squares regression is a method used for second-order data calibration, *e.g.*, excitation–emission matrices, and it consists of two steps. In EEM data modeling, the unfolding of the EEM spectrum into a data vector is first realized, and then a classical version of partial least squares (PLS) is applied for further data processing.^29^ Partial least squares is one of the most commonly used chemometric methods that allow the prediction of dependent variables (reference parameters) by determining a relationship between them and a set of independent variables, *i.e.*, measurement data that describes the samples. For this purpose, the new set of variables is designated (LVs, latent variables), which modeled this relationship by explaining maximum covariance between independent and dependent variables.^30^ Herein, the independent variables were 72 EEM spectra (6 exposure times × 12 replicants) unfolded into a data vectors of 1 × 1340 (1 × [*λ*_ex_ × *λ*_em_]), which were first arranged in a data matrix of 72 × 1340 (samples × [*λ*_ex_ × *λ*_em_]; see Data analysis section). The mean values of cells viability determined with the MTT assay were used as dependent variables, *i.e.*, 1-column target matrix. Before the UPLS model development, the data matrix of 72 × 1340 was randomly divided into train and test sets (75% and 25% of all data set, respectively). Therefore, the train matrix of 54 × 1340 was applied to establish the UPLS model, and an independent test matrix of 18 × 1340 was used for the external validation. The cross-validation of venetian blinds was used to determine the optimal number of LVs (based on the minimalization of Root Mean Square Error of Cross-Validation, RMSECV). The detailed evaluation of the quality of the determined model needs the comparison of the predicted values of cells viability with the reference values, *i.e.*, the predicted and actual values should be similar. Therefore, the performance of the UPLS model was characterized by linear fitting of the predicted values of cells viability to the reference values of cells viability (Fig. S1 in ESI†). In addition, to assess the quality of the obtained model, the parameters of linear fit (“*a*”, “*b*”, “*R*^2^”) were calculated for the train and test sets, assuming that in ideal model slope (“*a*”) is equal to 1, intercept (“*b*”) is 0 and determination coefficient (“*R*^2^”) is 1 (Table S1 in ESI†).

Based on the results obtained for the train set, it can be concluded that the developed UPLS model allowed for the determination of an appropriate relationship between the information on endogenous fluorophores encoded in the EEM spectra and the cells viability obtained in the MTT test. The satisfactory values of the linear fitting parameters confirm the high accuracy of the prediction at the training stage, *i.e.*, the value of the slope and the determination coefficient were both equal to 0.950 (Table S1†). The results obtained at the model validation stage using the test set are slightly worse, as evidenced by the decrease in the value of “*a*” and the coefficient “*R*^2^” (0.689 and 0.877, respectively; Table S1†). However, it should be noted that in the MTT assay, which was the reference method used to determine the viability of the cells, only the mean cells viability (±standard deviation) values for different UV irradiation times are obtained. Therefore, to check the compatibility of both methods, the analogous procedure of compiling the results of UPLS prediction was used as for the MTT test. Thus, the mean values of UPLS predicted cells viability and the standard deviation were calculated for each time point in the case of both train and test samples. The results obtained for both methods are summarized in Fig. 4. Moreover, to further investigate the accordance of the two presented methods, the statistical evaluation of the results was performed.

### Statistical evaluation of accordance of EEM fluorescence data and MTT test

The apparent similarity of the results obtained by MTT tests and EEM fluorescence spectroscopy is evident by studying Fig. 4. However, reliable verification of accordance of the two studied methods requires detailed statistical evaluation. First of all, the mean values of viability at respective time-points should not differ statistically significantly. To check if the null hypothesis about the equality of means can be rejected, for each time point *t*-test was performed based on the calculation of the experimental *t* value, which depends on the values of means, the observed standard deviations in the 2 studied methods, and the number of observations for respective time point. For the experimental value of *t* statistics, the *p*-value from *t* distribution was calculated and shown in Table 1 for each time point. *P*-value is the probability of obtaining test results at least as extreme as the obtained experimental results when the null hypothesis is true. Thus, using the standard value of significance level, *i.e.*, *α* = 5%, *p*-value higher than 0.05 implicates that the null hypothesis cannot be rejected. In other words, there is no statistical difference between the means obtained by the two studied methods of viability testing. For all time points, the calculated *p*-values were higher than 0.05 (results of *t*-test in Table 1). Therefore, the mean values of viability observed by the two methods, in each time point, are caused only by random errors. There is no systematic error and the high accuracy of EEM fluorescence outputs compared to the standard MTT test is proved, which shows high accordance of the two studied methods in terms of mean values.

The next step was to evaluate if the scatter of the results obtained in the MTT viability assay is comparable to this obtained by EEM fluorescence method, *i.e.*, accordance of precision level in the two methods was verified. For each time point, *F*-test was carried out based on the null hypothesis about the equality of variances. For the experimental value of *F* statistics, the *p*-value from *F*-distribution was calculated and presented in Table 1. As in the previous case, all *p*-values were higher than 0.05, which indicates that the null hypothesis cannot be rejected. In other words, the precision of the results obtained by the two studied methods does not differ statistically significantly.

**Table 1:** Comparison of the viability results obtained using MTT tests and EEM fluorescence analysis for various time points. *P*-values show no statistical differences obtained for each time point between mean values (accuracy) and standard deviations (precision). All *p*-values are higher than 0.05, indicating that there is no statistical difference between results obtained using the two methods (*α* = 5%)

	*t*/min					
	0	10	20	40	50	60
*F* test (precision)	1.7 × 10^−1^	1.7 × 10^−1^	5.1 × 10^−2^	1.5 × 10^−1^	1.0 × 10^−1^	4.4 × 10^−1^
*t* test (accuracy)	6.7 × 10^−1^	5.7 × 10^−2^	8.4 × 10^−1^	2.1 × 10^−1^	9.3 × 10^−2^	4.2 × 10^−1^

Viability tests are not usually applied to observe only one value of viability at the respective magnitude of stress factor, but rather they are used to trace the trend observed when it is increasing, *e.g.*, dose-depended effect in drug screening. Therefore in the next step we checked when the impact of UV radiation statistically significantly changes the viability. These calculations were again based on a *t*-test, but in this case, calculated for the same method but based on viabilities observed for various time points. Again, *α* = 5%, as a cut-off value for the null hypothesis, was applied. *P*-values obtained for the MTT test are presented in Table 2. All values greater than 0.05 (marked with green color) indicate that the observed difference is not statistically significant, whereas the statistical difference is observed in all other cases (marked with blue color, *p* ≤0.05). There are 3 groups of the results observed in the MTT test: “0 min” + “10 min”, which differs significantly from “20 min”, which also differs from the last group composed of the results obtained after expositions longer than 40 min (“40 min” + “50 min” + “60 min”). Therefore, the MTT test does not detect changes occurring after 10 min UV radiation (this time point is indiscernible from the control “0 min”), and after 40 min, the observed changes are negligible. At least these 3 groups should be visible when EEM fluorescence data is applied for viability studies. Thus, the same procedure was carried out for the fluorescence results, and the calculated *p*-values are shown in Table 3. In this case, much more of them are lower than *α* = 5%, *i.e.*, various time points are more discernable in terms of viability. 10 min exposure to UV radiation gives a statistically significant difference compared to the control (*p* = 4.4 × 10^−3^ in contrast to *p* = 5.6 × 10^−1^, compare Tables 2 and 3). As in the MTT test, viability observed after 20 min exposure is detectable and is discernable from longer expositions. Moreover, a statistically significant difference was also observed for 60 min irradiation compared to all lower time points. It must be underlined that the results obtained in “40 min” and “50 min” time points do not differ statistically significantly, but the same was observed in the MTT test (*p* = 2.6 × 10^−1^ and *p* = 5.6 × 10^−2^, respectively; both higher than 5%). Thus, in the EEM fluorescence test, even 5 groups of the results can be discerned: “0 min”; “10 min”; “20 min”; “40 min” + “50 min”; “60 min”. It means that EEM fluorescence spectroscopy is even more sensitive to viability changes than the MTT test; it can capture some subtle but detectable decreases of cells number.

**Table 2:** The results of *t*-tests as *p*-values showing significant (blue) and insignificant (green) differences between viabilities determined in MTT tests. In statistical terms, there is no significant difference between viability in “0 min” and “10 min” time points, as well as between “40 min”, “50 min”, “60 min” time points (*α* = 5%)

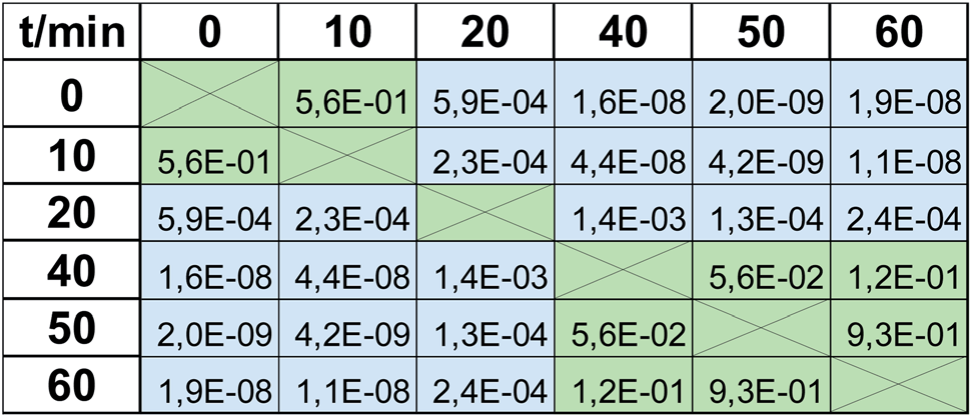

**Table 3:** The results of *t*-tests as *p*-values showing significant (blue) and insignificant (green) differences between viabilities determined by EEM fluorescence. In statistical terms, there is no significant difference only between “40 min” and “50 min” time points. Compared to Table 2, EEM fluorescence shows higher sensitivity – the results obtained for” 0 min” and “10 min” exhibit a statistically significant difference. The results obtained for ”60 min” are also discernible from the results obtained for “40 min” and “50 min” (*α* = 5%)

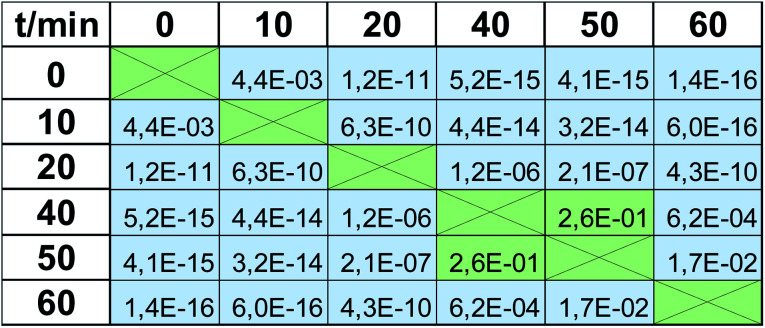

**Fig. 5:**
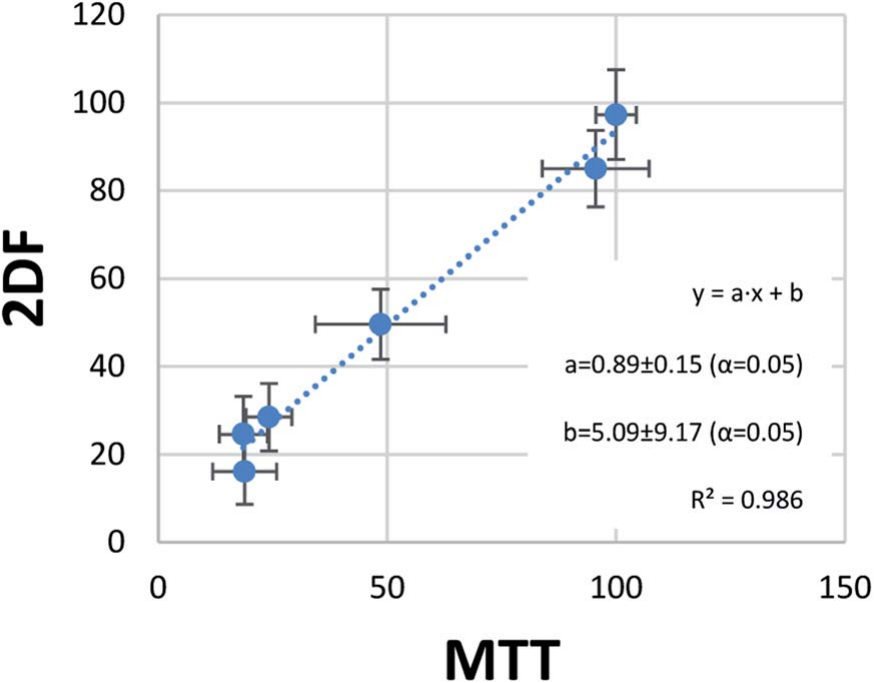
Correlation of viability results obtained with the use of MTT tests and EEM fluorescence analysis. Confidence intervals for “*a*” and “*b*” at *α* = 5% include the ideal values for such comparison (*y* = *x*, thus *a* = 1, *b* = 0), which indicate that the differences between mean results of MTT test and EEM fluorescence are only due to random errors (do not have statistical significance).

The last step of statistical evaluation included the study of the correlation between the results obtained by the two methods of viability testing. In one score plot, viability values obtained in the MTT test and EEM fluorescence were plotted, showing almost perfect correlation (Fig. 5). A very high determination coefficient (*R*^2^ = 0.986) indicates that only 1.4% of the variability in this data set is not explained by the linear fit. This very low value can be easily explained by random errors. The perfect linear fit should be obtained in perfect accordance with the results, which can be expressed with an equation *y* = *x* (*y* = 1 × *x* + 0). In our case, values of slope (“*a*”) and bias (“*b*”) were close to ideal values 1 and 0, respectively. Moreover, their confidence intervals obtained for confidence level 95% (*α* = 5%) revealed that they contained this ideal values of the perfect fit. In other words, *a* = 0.89 ± 0.15 does not differ statistically significantly from the ideal 1, and *b* = 5.09 ± 9.17 from the ideal 0 at *α* = 5%. This is another argument showing great accordance with the results obtained through MTT tests and the developed EEM fluorescence method.

### Avoiding pitfalls – the influence of UV radiation on cell culture medium

Excitation–emission matrix fluorescence spectroscopy is a non-selective technique, which means that the obtained EEM spectra might be affected by many factors influencing the fluorescence signal. In our case, the main interfering factor was the photodegradation of the components of the cell medium caused by UV radiation. Such a phenomenon can impact the obtained fluorescent landscape of the tested biological samples and adversely affect cell culture performance, leading to unreliable conclusions.^27^ Therefore, one of the critical aspects of this study was to check the influence of a selected stress factor on the EEMs of the cell culture medium. For this purpose, the additional series of experiments was carried out, in which the cell culture medium was subjected to the UV radiation for 0, 10, 20, 40, 50, 60 min in an analogous manner to the cell cultures (see Cell culture and reference data determination and materials section). Then the samples of the cell medium were incubated for 24 h, after which the EEM fluorescence measurements were conducted according to the measurement procedure used for cell culture (see The collection of EEM fluorescence data section). This step was crucial to select an appropriate methodology for cell culture preparation for fluorescence measurements to assess the actual possibility of monitoring cells viability using excitation–emission matrix fluorescence spectroscopy. Fig. S2† clearly shows that the excitation–emission matrices of the medium itself consistently changed during the exposure to UV radiation (see ESI†). Moreover, the UV irradiation of cell culture medium affected the same spectral regions in which the change of the fluorescence signal was observed in case of cell culture (around *λ*_ex_ ∈ (250 nm, 300 nm), *λ*_em_ ∈ (300 nm, 420 nm); *λ*_ex_ ∈ (300 nm, 340 nm), *λ*_em_ ∈ (360 nm, 460 nm); *λ*_ex_ ∈ (340 nm, 430 nm), *λ*_em_ ∈ (420 nm, 480 nm)). It should be noted that the photodegradation of the medium components resulted in far more visible alterations of the fluorescence signals than the subtle changes in the content of an individual, endogenous fluorophores in cell culture (Fig. 3 and S2†). From the perspective of chemometric data modeling, this means that failure to investigate this aspect adequately could lead to a mis-correlation of two completely unrelated effects. Therefore, to separate the effect of UV radiation on the medium from its influence on A375 cells, the cell culture medium had to be replaced immediately after UV irradiation. First of all, this procedure allowed us to ensure that the change in the composition of the medium under the influence of UV radiation does not affect the state of cell culture. Secondly, in this way, the observed differences in EEM spectra of A375 cells after 24 h of incubation in the fresh medium were related to changes in the content of endogenous fluorophores involved in the metabolic activity of the cells.

## Conclusions

Nowadays, the assessment of cell culture state can be performed through accurate and reliable tests based on differential staining or colorimetric assays. However, such methods become problematic when continuous, non-destructive monitoring is essential. Thus, spectroscopic techniques could be a solution to this issue, allowing for a non-invasive and label-free assessment of the state of cell cultures. In this work, we showed that EEM fluorescence fingerprints contain information correlating with cells viability. Moreover, this parameter can be estimated with the same accuracy and precision as in the “golden standard”, versatile but destructive, MTT viability assay. An additional advantage of EEM fluorescence softsensor is its compatibility with microplate readers, which enables high-throughput analysis.

However, information on cells viability included in EEM fingerprint is not accessible straightforward; it must be deconvoluted numerically using chemometric methods. As the chemometric model is built, it performs reliably only within its operational limits, and still, it is questionable if for other cell lines or other toxic factors it would also be useable. There is a need to make the proposed method more versatile, which will be the aim of our further studies.

## Conflicts of interest

There are no conflicts to declare.

## Supplementary Material

RA-012-D1RA09021F-s001
