# Supplementary material for: Excitation–emission matrix fluorescence spectroscopy for cell viability testing in UV-treated cell culture
Source: RSC Adv. 2022 Mar 9;12(13):7652–60. doi: 10.1039/d1ra09021f (PMC8982211; doi:10.1039/d1ra09021f)
Supplement: RA-012-D1RA09021F-s001 [file RA-012-D1RA09021F-s001.pdf]

Electronic Supplementary Material (ESI) for RSC Advances.
This journal is © The Royal Society of Chemistry 2022

Electronic Supplementary Information for

Excitation-emission fluorescence spectroscopy for cells viability testing in UV-treated cell culture

Klaudia Głowacz, Sandra Skorupska, Ilona Grabowska-Jadach, Patrycja Ciosek-Skibińska*

Chair of Medical Biotechnology, Faculty of Chemistry, Warsaw University of Technology, Noakowskiego 3, 00-664 Warsaw, Poland

*Correspondence: pciosek@ch.pw.edu.pl

Table of contents

Fig S. 1. .... **2**

Table S. 1. .... **2**

Fig S. 2. .... **3**

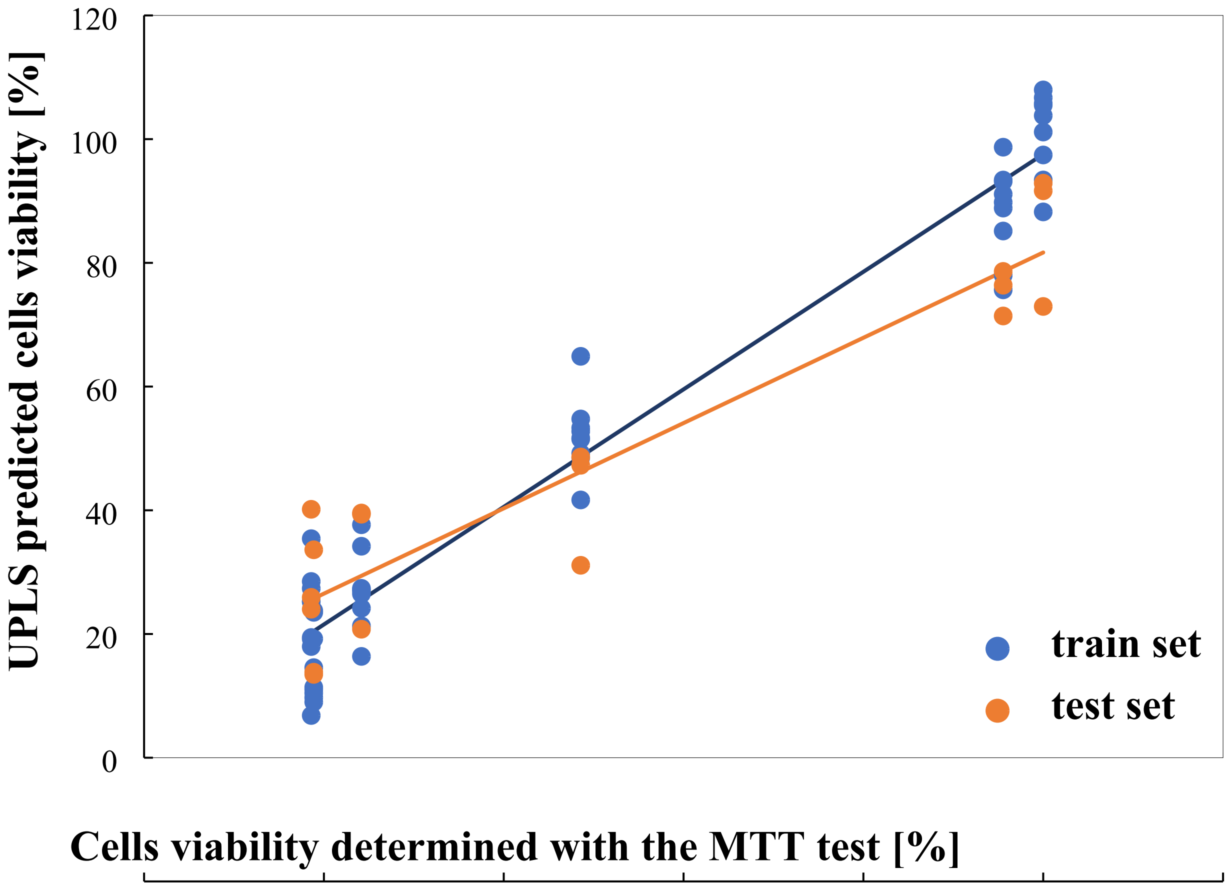

Fig S. 1. UPLS model performance shown as linear fit of the predicted cells viability vs. cells viability determined with the MTT test.

Table S. 1. Parameters of linear fit of UPLS-predicted cells viability and results of MTT test.

	**train set**	**test set**
**a**	0.950	0.689
**b**	2.535	12.737
**R^2**	0.950	0.877

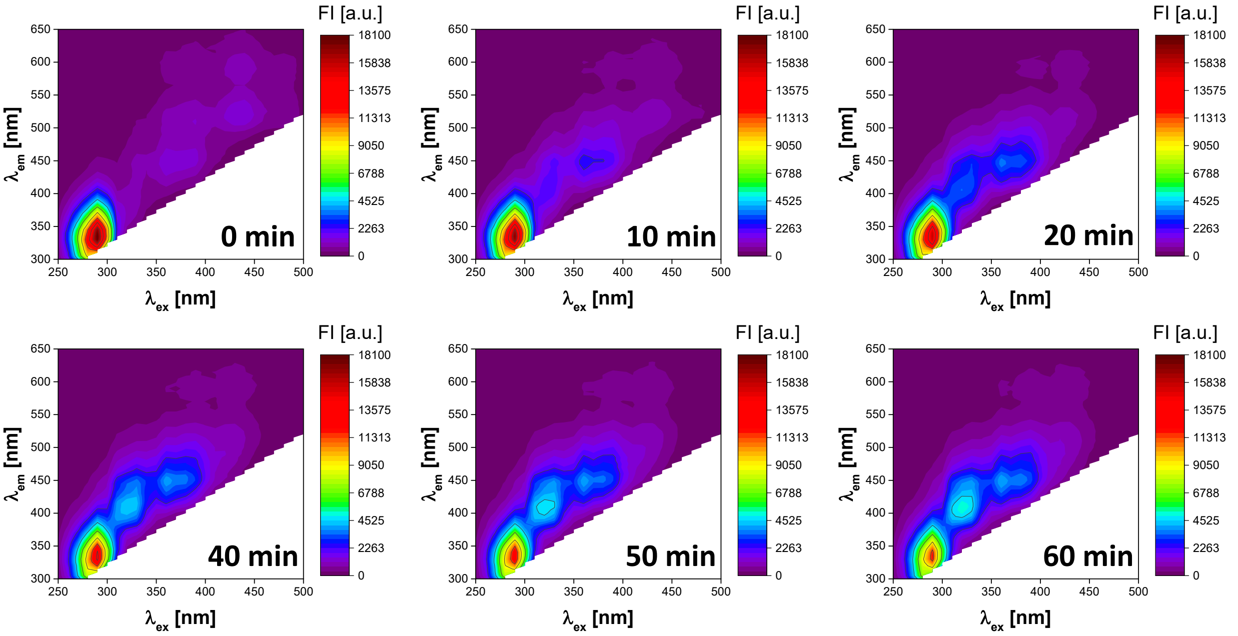

Fig S. 2. The influence of UV radiation of different time duration (0, 10, 20, 40, 50, 60 min) on excitation-emission matrix (EEM) of cell culture medium.
